# AGS Should Lead Through Education, Not Regulatory Advocacy: A Response to Social Determinants of Health Requirements

**DOI:** 10.1111/jgs.70032

**Published:** 2025-08-06

**Authors:** Richard G. Stefanacci

**Affiliations:** ^1^ Thomas Jefferson University, Jefferson College of Population Health Philadelphia Pennsylvania USA

**Keywords:** Beers Criteria, regulations, social determinants of health

The American Geriatrics Society's (AGS) recent advocacy to retain CMS social determinants of health (SDOH) data collection requirements, while well‐intentioned, misses an opportunity to demonstrate true leadership in geriatric medicine. Rather than defending federal mandates, AGS should champion the development and dissemination of evidence‐based best practices that empower clinicians to address social determinants effectively, independent of regulatory requirements [1, 2, 3].

The proposed CMS rollback of SDOH data elements and quality measures does not eliminate the clinical imperative to address housing, food security, and utility access—factors that profoundly impact our older adults' health outcomes. Experienced geriatricians have long recognized these social determinants as fundamental to comprehensive care, addressing them through clinical acumen rather than regulatory compliance.

The critical distinction lies between mandated data collection and meaningful clinical practice. While these regulations technically apply to hospitals, long‐term care hospitals, inpatient rehabilitation facilities, and skilled nursing facilities rather than individual clinicians, the practical burden of compliance falls squarely on the physicians, nurses, and other healthcare providers working within these institutions. Regulatory requirements do not implement themselves—they require clinicians to divert time from patient care to administrative documentation. Federal requirements often transform nuanced older adult conversations into standardized reporting exercises, diverting precious clinical time toward administrative tasks rather than direct care. This regulatory approach undermines the professional judgment that defines excellent geriatric practice.

Moreover, the argument that CMS requirements provide valuable opportunities to “capture” SDOH data reflects healthcare's fundamental flaw in prioritizing problem identification over problem resolution [4]. We have become exceptionally proficient at screening for social determinants—expertly identifying food insecurity, housing instability, and utility shutoffs using validated assessment tools. Documentation is thorough, quality metrics are satisfied, and continuing education requirements are met. Yet the underlying social needs of our older adults remain largely unaddressed, creating what amounts to an “assessment industrial complex” that produces excellent problem detectors but poor problem solvers. Rather than mandating additional data collection that duplicates this pattern, AGS should focus on developing and disseminating practical implementation strategies that actually address identified social determinant gaps. The goal should be solution competency, not surveillance proficiency.

AGS excels when it leads rather than follows. The organization's greatest contributions to geriatric medicine—most notably the ongoing development and refinement of the Beers Criteria for potentially inappropriate medication use in older adults—emerged from independent clinical leadership, not regulatory compliance. The Beers Criteria transformed geriatric prescribing practices worldwide precisely because they represented evidence‐based clinical excellence rather than bureaucratic mandates. Similarly, AGS's early advocacy for market‐driven development of assisted living facilities, emphasizing flexibility and consumer choice over rigid regulation, helped establish a high‐quality long‐term care option that now serves over 1 million older adults [5]. This market‐based approach created an alternative to the heavily regulated nursing home sector, which continues to struggle with quality concerns despite decades of federal oversight.

Conversely, AGS fails both its members and older adults when it subordinates its expertise to regulatory bodies or established organizations with competing interests. This pattern mirrors our organization's misguided 2009 strategy of encouraging members to join the AMA to secure influence within the Resource‐Based Relative Value Scale Update Committee (RUC). As my colleagues Drs. Mark Beers and Michael Wasserman and I argued then, “The AGS is seeking a seat at a table we have no business dining at.” [6] Rather than begging for representation within systems designed to disadvantage geriatrics, AGS should forge independent paths that advance our older adults' interests.

The parallels are striking: just as heavy regulation has failed to meaningfully improve nursing home quality while creating administrative burdens, and just as AMA control over physician reimbursement has systematically undervalued geriatric care, the current SDOH advocacy represents the same flawed thinking—legitimizing regulatory approaches that ultimately undermine clinical autonomy and professional judgment. Our most successful innovations in geriatric care have emerged from clinical leadership and market responsiveness, not federal mandates.

AGS should redirect its considerable expertise toward developing robust educational frameworks that teach clinicians how to identify, assess, and address social determinants effectively. This includes creating practical screening tools, intervention protocols, and community resource networks that can be adapted to diverse practice settings—from rural clinics to urban academic centers. More importantly, AGS should train members to effectively utilize existing population‐level SDOH data to inform their practice and advocacy efforts.

Rather than advocating for one‐size‐fits‐all federal mandates, AGS should establish voluntary certification programs, continuing education modules, and practice improvement initiatives that elevate the standard of geriatric care through professional excellence rather than regulatory compliance. Such an approach would respect regional variations in older adult populations, available resources, and practice environments while maintaining our commitment to comprehensive care.

*“Rather than advocating for one‐size‐fits‐all federal mandates, AGS should establish voluntary certification programs, continuing education modules, and practice improvement initiatives that elevate the standard of geriatric care through professional excellence rather than regulatory compliance.”*

The organization's influence should stem from clinical leadership and educational excellence, not from supporting federal micromanagement of clinical practice. Clinicians caring for older adults need practical guidance on implementing SDOH interventions and accessing existing data resources, not additional reporting requirements that burden already‐strained healthcare systems.

I urge AGS leadership to reconsider this position and instead focus on what we do best: advancing geriatric medicine through education, research, and the promotion of clinical best practices that improve older adult outcomes regardless of regulatory mandates.

## Author Contributions

Dr. Stefanacci was the sole author responsible for all aspects of this submission.

## Conflicts of Interest

The author declares no conflicts of interest.

## References

[1] American Geriatrics Society , “Comments on CMS‐1806‐P: Medicare Program; FY 2026 Hospital Inpatient Prospective Payment Systems for Acute Care Hospitals and the Long‐Term Care Hospital Prospective Payment System,” (2025), https://www.americangeriatrics.org/sites/default/files/AGS%20‐%20FY%202026%20IPPS%20NPRM%20Comments%20%28FINAL%29%206%2010%2025.pdf.

[2] American Geriatrics Society , “Comments on CMS‐1809‐P: Medicare Program; FY 2026 Skilled Nursing Facility Prospective Payment System,” (2025), https://www.americangeriatrics.org/sites/default/files/AGS%20‐%20FY%202026%20SNF%20NPRM%20Comments%20%28FINAL%29%206%2010%2025.pdf.

[3] American Geriatrics Society , “Comments on CMS‐1810‐P: Medicare Program; FY 2026 Inpatient Rehabilitation Facility Prospective Payment System,” (2025).

[4] R. G. Stefanacci , “From Problems to Solutions…The Need for the Right Education,” The Director, Summer/Fall, (2025).

[5] R. G. Stefanacci and P. M. Podrazik , “Assisted Living Facilities: Optimizing Outcomes,” Journal of the American Geriatrics Society 53, no. 3 (2005): 538–540. 15743303 10.1111/j.1532-5415.2005.53189_2.x

[6] R. G. Stefanacci , M. R. Wasserman , and M. H. Beers , “Moving Beyond the American Medical Association,” Journal of the American Geriatrics Society 57, no. 6 (2009): 1117–1118. 19490247 10.1111/j.1532-5415.2009.02293.x

